# Oil of Sweet Orange: A Possible Role in Carcinogenesis

**DOI:** 10.1038/bjc.1959.12

**Published:** 1959-03

**Authors:** F. J. C. Roe


### OIL OF SWEET ORANGE: A POSSIBLE ROLE IN CARCINOGENESIS

F. J. C. ROE

*From the Cancer Research Department, London Hospital Medical College, London E.1*

Received for publication December 13, 1958

OIL of sweet orange is obtained by expression from the peel of *Citrus sinensis*. It is imported into Great Britain from a variety of countries, but mainly from the U.S.A. The oil, or an essence prepared from it, is used as a food additive especially by the soft drinks, confectionery, and bakery trades ; it is also used pharmaceutically. The main constituents are limonene (> 90 per cent) and decanal. Citral, methylanthranilate, linalol and terpineol are present in some samples but not all, and there are probably yet further compounds present which have not so far been identified. These may include substances used to spray fruit against insect or other pests. The main constituent, limonene, is a monoterpene and does not contribute to the flavour of the oil. Decanal is the constituent mainly responsible for flavour.

The experiment reported here was part of a research project in which certain essential oils were to be tested for carcinogenic or co-carcinogenic activity for the skin of mice. In a preliminary test several oils (turpentine, lemon, peppermint, cinnamon, cloves, cedar wood) were applied to the skin of mice and biopsies taken 3 days later. Oil of sweet orange (B.P.C. grade), applied neat, was found to cause considerable epidermal hyperplasia. Moreover, the hyperplasia could be made to persist indefinitely and without concomitant ulceration by weekly applications of the neat oil or of an 80 per cent solution of the oil in acetone. None of the other oils tested gave rise to such a marked hyperplastic response. Oil of orange was therefore selected for test for carcinogenic and co-carcinogenic activity.

Forty male and 40 female mice of the " 101 " inbred strain maintained in this laboratory were divided into 4 groups of 20 mice each consisting of 10 males and 10 females. The first group were given a single application of 9,10-dimethyl-1,2-benzanthracene (DMBA), 300 $\mu$g. in 0·2 ml. acetone to the clipped dorsal skin, and no further treatment. The second group were given once weekly applications of 0·25 ml. neat oil of sweet orange (00). The third group received a single application of DMBA followed, after a 3 week interval, by weekly applications of neat 00. The fourth group were similar to the third except that instead of neat 00, weekly applications of 20 mg. urethane dissolved in 0·25 ml. 80 per cent 00 in acetone were given as the second treatment. The reason for the use of urethane in the 4th group is not relevant here, however it should be pointed out that previous results indicated that weekly applications of 60 mg. urethane in acetone after a single application of DMBA did not lead to tumour formation in " S " strain mice (Salaman and Roe, 1953).

The experiment is in its 24th week and all the mice are still alive. No papillomas are present in groups 1 or 2. In group 3, 9 mice have a single papilloma each ; and in group 4, 8 mice have a total of 15 papillomas. New papillomas are still appearing, and those already present are increasing in size. Seventeen out of the 24 are 2 mm. or more in diameter and the largest is 8 mm. In addition 1 mouse of group 3, and 1 of group 4 have developed haemangiomatous tumours in the subcutaneous tissue of the treated area. No malignant tumours of epidermal origin have so far been seen.

The experiment is being repeated and extended, and discussion will be deferred until the results of these further tests are known.

### SUMMARY

" 101 " strain mice painted with DMBA once followed by oil of sweet orange weekly developed many papillomas of the skin and one subcutaneous haemangioma. The addition of urethane to the oil of orange slightly increased the yield of tumours. Mice treated with DMBA only, or oil of orange only, developed no tumours during the same period. No malignant skin tumours have arisen so far (24 weeks).

The expenses of this research were partly defrayed out of a block grant from the British Empire Cancer Campaign.

### REFERENCE

SALAMAN, M. H. AND ROE, F. J. C.—(1953) *Brit. J. Cancer*, **7**, 472.

### ADDENDUM.

Since this paper was submitted for publication, malignant skin tumours have arisen in two mice of group 4, at 23 and 26 weeks respectively. On histological examination one of these tumours was found to be an undifferentiated carcinoma invading muscle: the other has not yet been examined histologically.


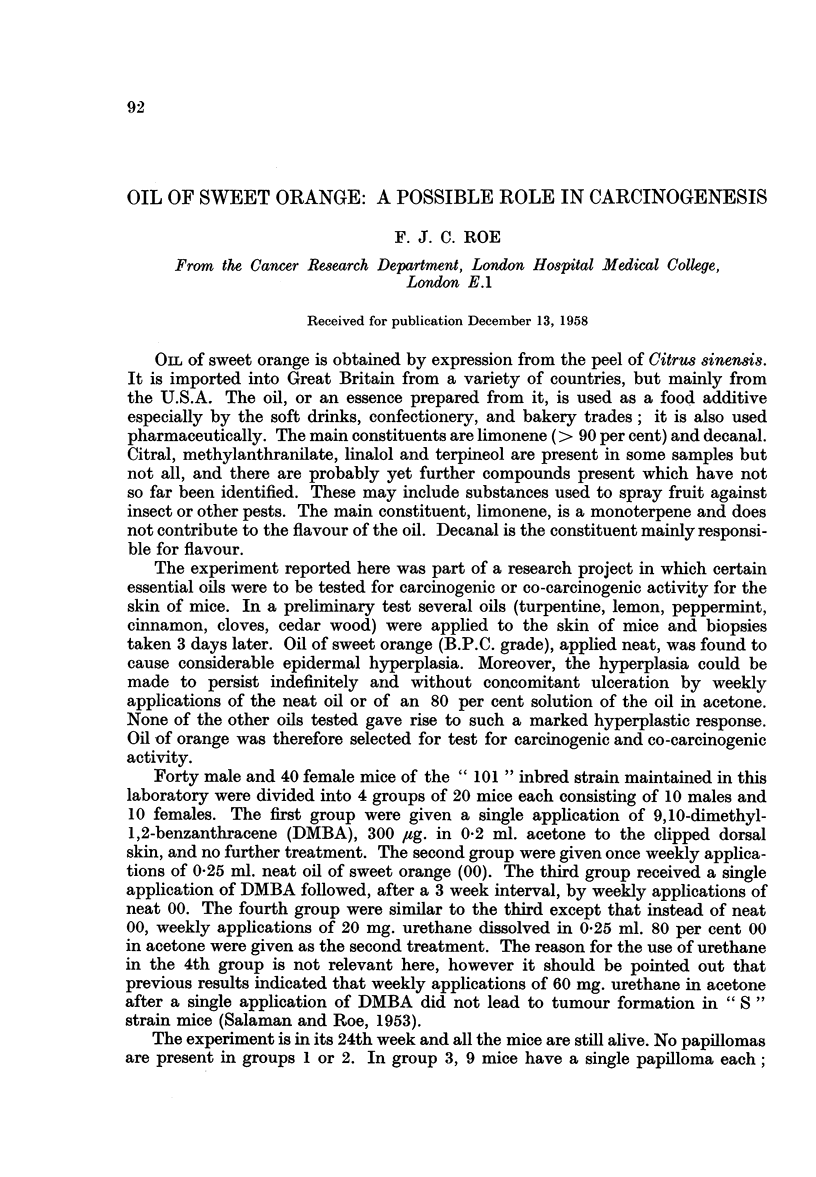

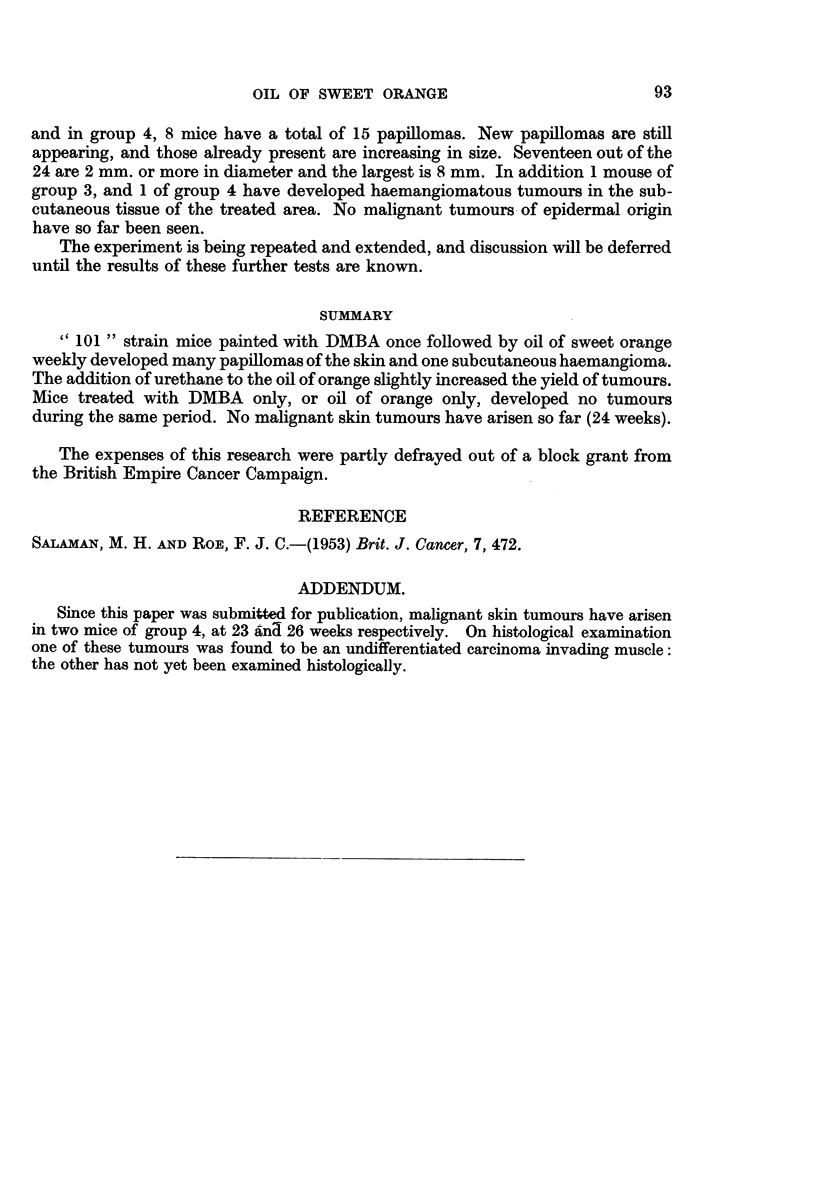


## References

[OCR_00081] SALAMAN M. H., ROE F. J. (1953). Incomplete carcinogens: ethyl carbamate (urethane) as an initiator of skin tumour formation in the mouse.. Br J Cancer.

